# Emergency Department Discharge Outcome and Psychiatric Consultation in North African Patients

**DOI:** 10.3390/ijerph15092033

**Published:** 2018-09-17

**Authors:** Osnat Keidar, Sabrina N. Jegerlehner, Stephan Ziegenhorn, Adam D. Brown, Martin Müller, Aristomenis K. Exadaktylos, David S. Srivastava

**Affiliations:** 1 Department of Emergency Medicine, Inselspital, Bern University Hospital, University of Bern, 3010 Bern, Switzerland; sabrina.jegerlehner@insel.ch (S.N.J.); stephan.ziegenhorn@insel.ch (S.Z.); martin.mueller2@insel.ch (M.M.); aristomenis.exadaktylos@insel.ch (A.K.E.); davidshiva.srivastava@insel.ch (D.S.S.); 2 Department of Psychology, New School for Social Research, New York, NY 10011, USA; brownad@newschool.edu; 3 Department of Psychiatry, New York University School of Medicine, New York, NY 10016, USA; 4 Institute of Health Economics and Clinical Epidemiology, University Hospital of Cologne, 50935 Cologne, Germany

**Keywords:** North African, immigration, health care, emergency department, disparities

## Abstract

Studies in Europe have found that immigrants, compared to the local population, are more likely to seek out medical care in Emergency Departments (EDs). In addition, studies show that immigrants utilize medical services provided by EDs for less acute issues. Despite these observed differences, little is known about the characteristics of ED use by North African (NA) immigrants. The main objective of this study was to examine whether there were differences in ED discharge outcomes and psychiatric referrals between NA immigrants and Swiss nationals. A retrospective analysis was conducted using patient records from NA and Swiss adults who were admitted to the ED of the University Hospital in Bern (Switzerland) from 2013–2016. Measures included demographic information as well as data on types of admission. Outcome variables included discharge type and psychiatric referral. A total of 77,619 patients generated 116,859 consultations to the ED, of which 1.1 per cent (*n* = 1338) were consultations by NA patients. Compared to Swiss national patients, NA patients were younger, with a median age of 38.0 (IQR 28–51 years vs. 52.0 (IQR 32–52) for Swiss and predominantly male (74.4% vs. 55.6% in the Swiss). NA patient admission type was more likely to be “walk-in” or legal admission (7.5% vs 0.8 in Swiss,). Logistic regressions indicated that NA patients had 1.2 times higher odds (95% CI 1.07–1.40, *p* < 0.003) of receiving ambulatory care. An effect modification by age group and sex was observed for the primary outcome “seen by a psychiatrist”, especially for men in the 16–25 years age group, whereby male NA patients had 3.45 times higher odds (95% CI: 2.22–5.38) of having being seen by a psychiatrist. In conclusion differences were observed between NA and Swiss national patients in ED consultations referrals and outcomes, in which NA had more ambulatory discharges and NA males, especially young, were more likely to have been seen by psychiatrist. Future studies would benefit from identifying those factors underlying these differences in ED utilization.

## 1. Introduction

According to the International Organization for Migration (IOM) a migrant refers to any individual that moves across international borders away from her or his country of origin, regardless of legal status or cause [1]. Current socio-economic, environmental, and political forces in recent years has led to an unprecedented number of people migrating from low and middle-income nations to high-income countries, such as Switzerland [2,3]. Although people are immigrating to Switzerland from a number of geographical regions, a high proportion of individuals are coming from Africa [4]. Recent estimates suggest that approximately 6.7% of all foreign residents come from African countries [5]. More specifically, among those individuals migrating from Africa, a majority are from North African (NA) countries such as Morocco, Egypt, and Tunisia [6]. Supplementary Figures S1 and S2 present the population structure by age groups in male and female for NA and Swiss populations.

Although people from NA represent a small proportion of all individuals migrating to Switzerland, Switzerland has recently seen a rapid rise in migration from NA countries, which has, in part, led to Swiss policy makers pursuing cooperation programs between Switzerland and NA countries on issues of migration and protection [7]. Despite the focus on immigration policy and security issues, not much research, to date, in Switzerland has begun to examine potential health care needs among this population.

Despite the lack of data on the health care needs of individuals from NA in Switzerland, migrants, including those from NA, that arrive to Europe, are at risk for various infection and non-communicable diseases (NCSs). For example, migrants have an increased risk for cardiovascular diseases than the population of the host country [8,9], and in NA migrants in particular a higher risk for Hepatitis B and C, as well as HIV, among other infectious diseases, is reported [10]. Studies also suggest that migration from various countries, including NA, is associated with trauma and greater likelihood of mental health issues [11,12].

One area that might be of particular importance to NA patients, as one station in addressing their health needs, is the utilization of care in Emergency Departments (EDs). In general, European countries, including Switzerland, have reported an increased utilization of EDs among patients with asylum-seeking status [13]. Furthermore, studies have identified a number of important differences in the ways in which immigrants in Europe seek care in EDs [14]. For example, immigrants were more likely to go to the ED for non-acute issues [14,15] and during less “social hours” (e.g., evenings and weekends) [14]. Other studies have reported that the high use of emergency services may be related to inadequate levels of health literacy, a lack of health care system knowledge, lack of access to a general practitioner, undocumented immigration status and language barriers [16,17,18,19,20].

Although migration in and of itself has not been always associated with poor health, the physical, psychological, economic, and social challenges associated with migration have been associated with the presence of psychiatric symptoms. Given the documented challenges that NA migrants face in their country of origin as well as known stressors associated with migration, individuals from NA may be more likely to be in need of psychiatric care in the ED. However, mental health problems in the ED among migrants in general is sparse, and even less is known about NA migrants in particular. Therefore, given the well-documented stressors and exposure to potentially traumatic events associated with migration [21], there is an urgent need to identify whether there are differences between immigrant and non-immigrant utilization of ED resources. If found, such data would point to an important point in the detection and intervention of mental health care services.

To that end, the aim of this study was therefore: (1) to compare the types of admissions to the ED among NA and Swiss-national patients, (2) to examine potential differences in referrals to ambulatory care and psychiatry between NA and Swiss national patients and (3) to identify factors associated with referrals and to ambulatory care and psychiatry. As migration from NA countries is challenging European countries, understanding these aspects should not be a local need, but rather can contribute to the better utilization of healthcare services across the continent, with an aim of improving their health through the implementation of such findings into interventions to improve access and care.

## 2. Methods

### 2.1. Design

#### 2.1.1. Setting

The data for the current study was obtained through a retrospective analysis of patient records that had been admitted to the ED of the University Hospital of Bern, Switzerland. It is a level 1 adult ED caring for about 2 million people and in which approximately 46,000 people were treated in 2016 [22]. All adult patients (age ≥ 16 years) admitted to the ED between the 1 January 2013 and the 31 December 2016 were included.

#### 2.1.2. Participants

Patients were identified through the ED electronic patient database and were separated into two groups: (1) patients of NA origin and (2) Swiss nationals. All patients 16 years of age and older were included. According to the Swiss law, the cutoff for treatment in general adult ED is 16. Patients younger than sixteen are referred to the pediatric ED. Exclusion criteria included patients aged less than 16 years, treated in the pediatric ED and with missing documentation of country of origin, triage or leading referred discipline.

### 2.2. Data Collection

Patients were identified through the ED electronic patient database (E-Care, ED 2.1.3.0, Turnhout, Belgium) and exported into Microsoft Excel (Microsoft Corporation, Redmond, WA, USA) after anonymizing the data.

#### Variables

Socio-demographic variables included country of origin, determined through the resident card presented at admission. Patients were considered to be from NA if their country of origin was Algeria, Egypt, Libya, Morocco, Sudan or Tunisia. Other variables included type of ED admission (ambulance, walk-in, legal admission [police custody or presence], previous medical contact, and not specified), discipline seen in ED (internal, surgical, psychiatric, other), discharge outcome (ambulatory, hospitalized, death, other), triage level (Swiss triage scale) (1 = life threatening problem that requires an immediate start of treatment to 5 = not urgent condition). The Swiss triage scale is based on the Manchester triage scale which is a five- level scale that was tested for its reliability and validity [23], patient receives an appointment), time of admission (weekday or weekend, where a weekend visit was determined as arrival to ED between Friday 18:00 to Monday 07:00), length of stay in ED (number of hours), and number of ED visits per patient. Ambulatory discharge was determined as being discharged home after the consultation. Seeing a psychiatrist included all ED consultations in which a psychiatrist was the prime or a secondary consultant.

### 2.3. Statistical Analysis

Statistical analysis was conducted using SPSS Version 22 (BM SPSS Statistics for Windows, IBM Corp., Armonk, NY, USA) and Stata 13.1 (StataCorp, College Station, TX, USA). Descriptive calculation included frequency and percentage of categorical data as well as median and IQR for all continuous variables, as all (age, time in ED and number of visits) had non-normal distribution. Statistical significance was defined as *p* value < 0.05. For the determination of predicting factors for the two outcome variables (ambulatory discharge and psychiatric consultations), an adjusted logistic regression was conducted. These results ware compared to a model that looked at effect modification for the primary outcomes (ambulatory discharge and psychiatric consultation) by age and sex through an interaction model with presentation of the stratified odds ratios. Age groups were defined as 16–24 [24] (youth promotion measures in Switzerland being aimed at youth aged 16–25 years), 25–39 (young adulthood), 40–64 (later adulthood) and ≥65 (retiring age in Switzerland). As effect modification was identified, only the results of the interaction models were presented for both outcome variables.

## 3. Results

**Figure 1:**
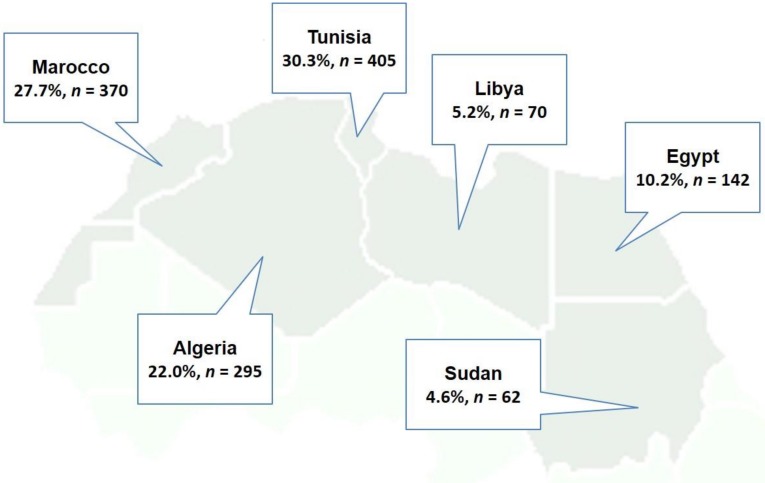
Frequencies of countries of origin of the North African migrant ED population.

During the study period, a total of 77,619 Swiss national and NA patients (76,889 Swiss, 98.9%) cumulatively generated 116,859 visits to our ED, with 1338 (1.1%) originated from NA and the others with Swiss nationality. The most common country of origin was Tunisia (405 patients, 30.3%), followed by Morocco (370 patients, 27.7%), Algeria (295 patients, 22.0%), Egypt (142 patients, 10.2%), Sudan (62 patients, 4.6%), and Libya (70 patients, 5.2%) (Figure 1). 

### 3.1. Comparison between NA and Swiss Origin ED Patients’ Consultations Characteristics

There were some observed demographic differences between patients from NA and the Swiss population (Table 1). Specifically, patients originating from NA countries were predominantly male (74.4% vs. 55.6% in the Swiss) and younger (median of 38.0 (IQR 28–51 years vs. 52.0 (IQR 32–52) for 154 Swiss). The differences in patient population at discharge reflect the starkly different demographic characteristics of the two populations in our study region (see Figures S1 and S2).

More than half of the visits were walk-in emergencies with the NA patients having significantly more walk-in presentations than the Swiss (55.5% vs. 42.2%). NA patients were assessed in a less acute triage level than the Swiss patients with 79.6% in triage 3 or higher compared to 65.9% in Swiss nationals patients. Additionally, NA patients were more likely to receive an ambulatory care discharge compared to patients from Switzerland (64.2% and 47.4% respectively,) and had shorter length of stay in the ED (median 3.4 h (RQI 2.0–5.4 years) for NA vs 3.6 for Swiss (IQR 3.2 and 3.6 respectively,). However, the median of total visits number was 2.0 for both groups, IQR 1–4 visits for NA and 1–3 for Swiss), with more NA patients having multiple visits (45.4% for NA vs. 33.4% for Swiss). When looking at reasons for admission, the NA patients had higher proportion of admission for general surgical, medical, and psychiatric reasons. In contrast, Swiss patients had higher proportions of admission to specialists (Table 1). 

### 3.2. Predictors of Ambulatory Discharge

A logistic regression was performed to determine the effects of possible predicting variables on the odds of being discharged to ambulatory care. NA patients had 1.2 times higher odds (95% CI 1.07–1.40, *p* < 0.003) of being treated as an out-patient in comparison to Swiss national patients, adjusted for age, sex, triage, weekend admission, discipline, weekend consultation and type of admission. To examine effect modification in age groups and sex, an interaction model was conducted. Results are presented in Table 2. 

The CI of the odds ratios differed in some of the different age-sex-strata, e.g., in the 40–64 group NA patients were associated with greater odds of ambulatory discharge, but not in the 25–39 age group. Thus, the studied effect is modified by age and sex respectively and their interaction and stratified odds ratios have to be used (Table 2).

### 3.3. Predictors of Psychiatry Referral

To further understand resource utilization in the ED, a second model was run examining the predictors of psychiatric consultation between NA and Swiss national patients. A logistic regression was conducted. Consultations by NA patients had a 1.75 (95% CI: 1.5–2.0, *p* < 0.0001) times higher odd of being seen by a psychiatrist adjusted for age, sex, triage, weekend visits, and multiple ED visits than consultations by Swiss patients. Again, to examine effect modification of age groups and sex, an interaction model was applied. Results are presented in Table 3.

There are noted differences in some of the strata between males and females. The odds ratio for seen by psychiatrist in females aged 25–39 years (OR 0.51, 95% CI 0.27–0.94), suggested evidence for a “protective” effect of being NA in adult females regarding being seen by a psychiatrist. In contrast, among males, being NA increased the odds of being seen by a psychiatrist about the factor 2 (OR 1.95, 95% CI 1.53–2.49). Thus, the effect of the association between the exposure (NA vs. Swiss) and the studied outcome (seen by a psychiatrist) is strongly modified by sex, and in the interaction of sex and age. Especially, sex is a strong effect modifier. For age, there seems to be a slight effect modification too. The odds ratio for seen by a psychiatrist in the very young (age 16–24) NA male group is about 3.5 times higher (OR 3.45, 95% CI 2.22–5.38) in comparison with the Swiss national males of the same age, whereas in the young adults (age 25–39) male group the odds ratio point of higher risk was almost twice (OR 1.95, 95% CI 1.53–2.49) in comparison to Swiss national males in the same age (Table 3).

## 4. Discussion

This is the first study, to our knowledge, comparing the utilization of ED services and referrals in NA immigrants and non-immigrants in a high-income country. Specifically, this study sought to examine whether NA immigrants and non-immigrants would differ in their frequency of ED use and referral type during their ED visit and upon hospital discharge. Converging with a growing body of research showing that immigrants and non-immigrants differ in their utilization of ED services, these data found that NA patients differed from Swiss national patients in a number of important ways. First, the demographic characteristics differed between NA and Swiss-national patients. That is, NA ED patients were more likely to be male and younger. Second, compared to Swiss national patients, NA patients were more likely to seek care for less acute issues, had greater number of total visits and re-visits, and spent fewer hours in the ED [15,25]. 

Additionally, a greater proportion of NA patients arrived at the ED through self-referral or through a legal context, whereas Swiss national patients were more likely to have been referred to the ED through another healthcare provider. Third, the two groups differed in terms of referrals and discharge type: NA patients were more likely to see a psychiatrist in the ED and were more likely to be discharged to ambulatory care. 

Taken together, these findings begin to shed light on the importance of examining immigrant communities use of healthcare resources within the ED, as it reveals the changes in how patients are using the ED and possibly the healthcare needs. Importantly, these data underscore the importance of studying how immigrants are using the ED, as immigrants appear to be seeking care for non-urgent issues in this setting, rather than through a primary care provider and consecutively generate more visits and lower hospitalization rates than non-immigrant patients. 

It is unclear from these data why NA patients are using the ED more often and for less urgent matters. Findings from other studies, however, suggest that immigrants may be more likely to seek care in EDs, compared to seeing other medical specialties, for a variety of legal, cultural, and social factors [26]. In addition, some studies have found that lower levels of healthcare literacy in immigrant communities may impact medical decisions [20,27,28]. Third, although not necessarily the case in Switzerland, immigrants often receive minimal coverage through their insurance, and in some cases, may only receive insurance for emergencies [15]. Therefore, it may be perceived in some immigrant communities that they may not be eligible for care outside the ED. Given this growing number of studies showing a disproportionally greater use of the ED among immigrants, further research is needed to better understand the motivations underlying ED use and barriers to care among other medical specializations. 

The greater utilization of the ED among NA patients, both in terms of total and multiple visits, for non-acute medical issues suggests that there is an important need for healthcare systems to consider ways to reduce patient visits. These findings point to the need to develop healthcare literacy programs targeting ED use. A number of community-based strategies for increasing health literacy have proven effective [29,30]. A possible approach for a comprehensive intervention that are hoped to increase patient engagement is the use of the social-ecological model [31]. Future work would benefit from examining whether similar programs may aid in the reduction of ED visits and re-visits for non-acute issues. 

Importantly, NA patients in this study were more likely to receive a referral for psychiatric care. Unfortunately, specific mental health disorders were not assessed in this study so the exact cause for the referral is not known. These findings, however, are directly in line with considerable research showing the immigrants are exposed to considerable stress and trauma throughout the migration process, which has been associated with high levels of mental health issues such as depression and PTSD [32,33]. Future work would also benefit from examining the extent to which the patients received the follow-up psychiatric care. Although speculation, one potential reason for the multiple visits, is that the NA patients, for a variety of reasons (e.g., language barriers, lack of trust, cultural beliefs), may not have been enrolled into on-going psychiatric care, and instead continued to seek help from the ED. 

These data also point to the potential importance of incorporating brief mental health interventions for immigrants into ED. Given the high rates of psychiatric referrals, offering brief psycho-social interventions within the ED may aid in the reduction of distress and may help to motivate patients to seek additional care. Studies indicate that culturally adapted mental health interventions have a higher potential of being effective, with a focus on groups of same background and in patients’ language [34]. An example for such intervention is the International Psychosocial Organization (IPSO) psychos-social counselling program targeted to refugees. The program trains counsellors within the community, to enable a linguistic and culturally sensitive service [35,36]. Future work needs to examine whether similar programs can be integrated into an ED context, for patients identified with mental health problems. Along those lines, these findings indicate that medical staff in the ED might benefit from training in this area as many immigrants may present with complaints that include mental health symptoms. Such training would benefit from the inclusion of culture competence capacity building for physicians and nurses, with existing evidence on the effectiveness among trained professionals in a hospital setting in Switzerland [37]. Similar training was conducted in our ED by the Swiss Red Cross.

It is unclear why NA patients were more likely to arrive in the ED from legal contexts. It may be related to co-occurring and improperly managed mental health concerns, stressors associated with post-migration (e.g., low socio-economic status), and/or potential selection biases in which the police may be more likely to bring an NA patient to the ED in unclear situations. This has the potential to create a vicious circle that leads to lower quality of care and again to readmissions. 

Despite the novel contributions of these data to the understanding of how immigrants in the ED, several limitations warrant discussion. First, it is a retrospective study. Follow-up research would benefit from clinical interviews and the employment of prospective methodologies. Second, as previously, mentioned, these data indicate the type of referral but not the specific diagnosis. Also, data on morbidity is not available. Therefore, greater work is needed to better characterize the issues being presented in the ED. Moreover, we cannot provide information on medical condition of pregnant women and children, as they are usually treated at different EDs within our hospital. Lastly, it would be beneficial to stratify in our analysis the Swiss patients into socioeconomic groups, in order to assess if NA population is more closely matched to a particular socioeconomic Swiss group. Unfortunately, this data was not available in our ED records and such analysis could not be conducted.

Notwithstanding these limitations, these data emphasize the importance of the ED in the care of recent immigrants. In particular, they point to an evolving use of the ED in which patients are seeking care more regularly for less acute issues. As studies provide more information on the underlying factors contributing to these patterns, health care providers will need to consider ways to target healthcare literacy more effectively and leverage the types of care provided by ED for immigrants. These findings may be applicable also outside of Switzerland, as the immigration from NA is a continuous phenomenon across Europe, and the culturally sensitive interventions that address these challenges, can serve as a major contribution to a better utilization of ED resources and assist in improving the health of NA migrants. One framework that might be good for integration of such programs across Europe is the Health Promoting Hospitals and Health Services network, that uses the healthy settings approach in an aim to integrate health promotion concepts, values, standards and indicators into the organizational structure and culture of the hospital of the health service, to gain better health to patients, staff and communities. The initiative includes a focus on Migrant friendly and culturally competent health care [38]. In Switzerland, in particular, the “Swiss Hospitals for Equity” network, where our ED is partnering in the activities, aims to improve health and health services to migrants in the hospital setting [39].

## 5. Conclusions

The study identified differences between the ED patients from Switzerland and NA. NA patients were less likely to be admitted to the hospital and NA males were more likely to be referred to a physiatrist. NA male patients are more likely to be seen by psychiatrist when they seek consultation at the ED, especially in very young adults (16–24) suggesting that those are of special need for special attention and further follow-ups as needed. The different patterns of ED care use between Swiss nationals and NA migrants, were associated with significantly more ambulatory discharges stress the need to focus on ways to ensure access to primary health services to migrant population. Measures that promote equity are paramount in the different population presented to the ED. On the patient level, interventions that focus on case management for patients with re-visits [40] are also recommended, with a hope that using multiple-dimensions approaches could support in slowly closing the health gaps between the two populations.

## Figures and Tables

**Table 1:** Characteristics of ED consultations—comparison between Swiss and NA nationals consultations (*n* = 116,859).

	NA Nationals		Swiss National	
Total, *n* (%)	1338	1.1	115,521	98.9
Gender				
Male	996	74.4	64,250	55.6
Female	342	25.6	51,271	44.4
Reason for admission, *n* (%)				
Surgical	425	31.8	24,756	21.4
Medical	412	30.8	30,637	26.5
Psychiatric	102	7.6	5258	4.6
other discipline	399	29.8	54,870	47.5
Way of admission, *n* (%)				
Ambulance	155	11.6	19,305	16.7
previous medical contact	55	4.1	18,104	15.7
legal admission	101	7.5	946	0.8
walk-in	743	55.5	48,772	42.2
Other	48	3.6	4609	4.0
Triage, *n* (%)				
1 (life threatening)	42	3.6	10,386	9.0
2 (urgent conditions)	232	17.3	28,942	25.1
3 (semi-urgent conditions)	909	67.9	66,308	57.4
4 (low urgent conditions)	113	8.6	7531	6.5
5 (not urgent conditions)	42	3.1	2354	2.0
ED outcome, *n* (%)				
discharge at home	859	64.2	54,989	47.6
hospital admission	268	20.0	41,508	35.9
Death	0	0.0	222	0.2
Not specific	170	12.7	17,510	15.2
Other	41	3.1	1292	1.1
Weekend admission, *n* (%)	437	32.7	39,137	33.9
Multiple visit, *n* (%)	306	45.4	38,632	33.4
Seen by psychiatrist, *n* (%)	193	14.4	8417	7.3
Age, med (IQR)	38.0	28–51	52.0	32–52
Duration in ED (hours), med (IQR)	3.4	2.0–5.4	3.6	2.2–6.2
Number of visits, med (IQR)	2.0	1.0–4.0	2.0	1.0–3.0

**Table 2:** Sex and Age group stratified odds ratios * (95% CI) for the outcome ambulant discharge for the comparison NA vs. Swiss (*n* = 116,859). (significant associations in italic and bold).

	Age Group			
**Sex**	**16–24**	**25–39**	**40–64**	**65–Max**
Female	0.89 (95% CI: 0.38–2.06)	1.45 (95% CI: 0.96–2.19)	***1.56 (95% CI: 1.01–2.39)***	3.31 (95% CI: 0.93–11.76)
Male	0.94 (95% CI: 0.61–1.46)	1.02 (95% CI: 0.81–1.27)	***1.28 (95% CI: 1.01–1.63)***	1.78 (95% CI: 0.76–4.14)

* adjusted for type of admission, triage category, discipline category, multiple consultation, weekend consultation.

**Table 3:** Sex and Age group stratified odds ratios * (95% CI) for the outcome seen by psychiatrist for the comparison NA vs. Swiss (*n* = 116,859) (significant associations in italic and bold).

	Age Group			
**Sex**	**16–24**	**25–39**	**40–64**	**65–Max**
Female	1.25 (95% CI: 0.48–3.24)	0.51 (95% CI: 0.27–0.94)	0.68 (95% CI: 0.36–1.3)	2.81 (95% CI: 0.37–21.64)
Male	***3.45 (95% CI: 2.22–5.38)***	***1.95 (95% CI: 1.53–2.49)***	***1.94 (95% CI: 1.44–2.61)***	2.98 (95% CI: 0.7–12.68)

* adjusted for triage, multiple consultation, weekend consultation.

## Supplementary Materials

The following are available online at http://www.mdpi.com/1660-4601/15/9/2033/s1, Figure S1: NA population in Canton Bern by age groups and gender (%), Figure S2: Swiss population in Canton Bern by age groups and gender (%).

## Availability of Data and Material

The datasets used and/or analysed during the current study are available from the corresponding author on reasonable request.

## Abbreviations

ED	Emergency Department
HPC	Health care providers
NA	North Africa
CDC	Non Communicable Disease
IPSO	International Psychosocial Organization
