# Supplementary material for: Emergency Department Discharge Outcome and Psychiatric Consultation in North African Patients
Source: Int J Environ Res Public Health. 2018 Sep 17;15(9):2033. doi: 10.3390/ijerph15092033 (PMC6163756; doi:10.3390/ijerph15092033)
Supplement: Supplementary file 1 [file ijerph-15-02033-s001.pdf]

Supplement material

Figure S1: NA population in Canton Bern by age groups and gender (%)

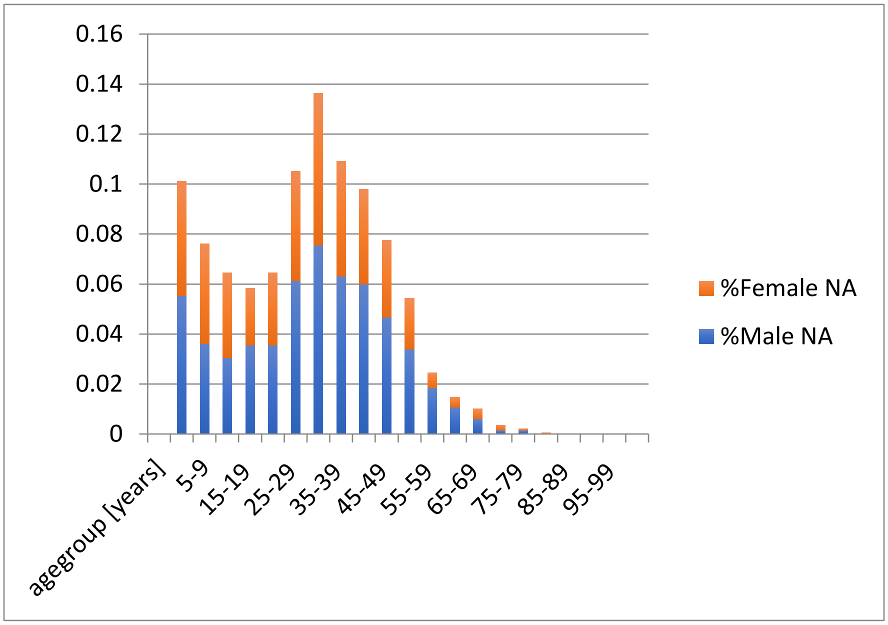

Figure 2: Swiss population in Canton Bern by age groups and gender (%)

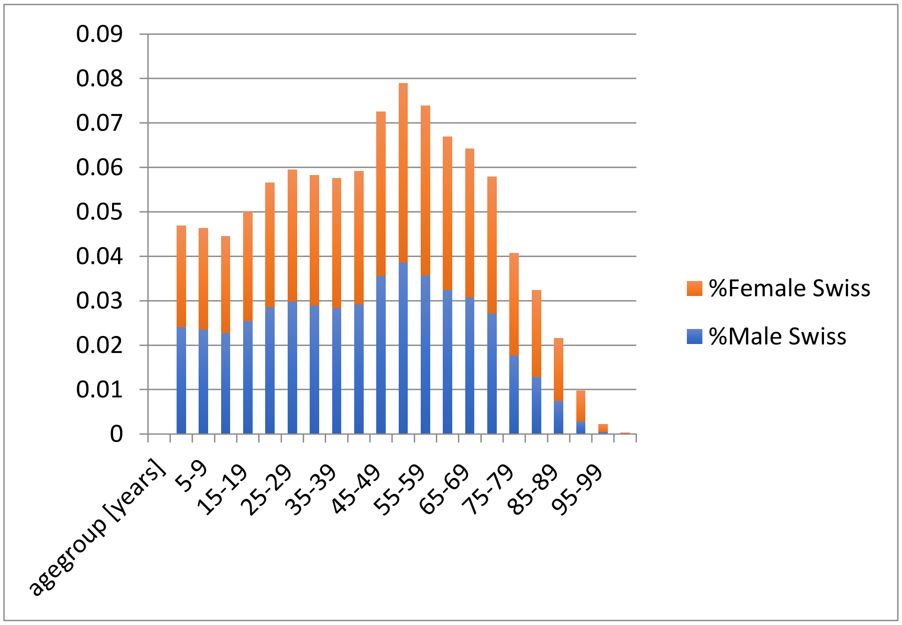

Source: Swiss Federal Office of Statistics, Statistical Yearbook of Switzerland

https://www.bfs.admin.ch/bfs/de/home/statistiken.html
